# The Impact of Pet Videos on Emotional Face Processing

**DOI:** 10.3390/ejihpe16020021

**Published:** 2026-02-09

**Authors:** Xingyu Zhu, Xiaojing Shi, Jiahao Liang, Bukuan Sun, Wuji Lin, Jingyuan Lin

**Affiliations:** 1 Institute of Brain and Psychological Science, Sichuan Normal University, Chengdu 610066, China; 2 School of Education, Fujian Polytechnic Normal University, Fuzhou 350300, China

**Keywords:** pet video, emotion regulation, attention bias, emotion perception, emotional face

## Abstract

In recent years, the number of people viewing pet videos and images online has risen. Although numerous studies have shown that owning pets positively impacts human mental health, the potential mental health benefits of prolonged exposure to pet media content remain debated. This study conducted three experiments to investigate how viewing pet videos affects human emotional face processing and to clarify the associated emotional regulatory mechanisms. Experiment 1 examined how viewing pet videos influences attentional bias toward emotional faces. Experiment 2 assessed the impact of watching pet videos on the valence perception of emotional faces. Experiment 3 analyzed how exposure to pet videos affects the valence perception of emotional text. The results showed that watching pet videos increased attentional bias toward subsequent positive emotional faces and decreased bias toward negative ones. This effect resulted from higher perceived valence ratings for both positive and neutral emotional faces. Importantly, this effect was only observed in facial stimuli with social attributes. These findings indicate that watching pet videos modulates emotional processing, and prolonged exposure to pet media content may affect mental health through this mechanism.

## 1. Introduction

The beneficial effects of pets on human physical and mental health have been substantiated by numerous studies (see review Wells, 2019; Liu et al., 2024). Pets are frequently recognized as vital sources of emotional support, with their presence demonstrating significant improvements in human emotional states (Janssens et al., 2021) and modulation of emotion-related neural activity along with alterations in neurotransmitter secretion (Liu et al., 2024, 2025). Cumulative evidence has established pets’ therapeutic roles in stress reduction (Junça-Silva et al., 2022; Janssens et al., 2021; Rathish et al., 2022), anxiety alleviation (Kogan et al., 2021; Shearer et al., 2016; Hoagwood et al., 2022), depression mitigation (Brooks et al., 2018; Cheung & Kam, 2018; Hajek et al., 2024), and life satisfaction enhancement (Junça-Silva, 2022; Quan et al., 2023). Recent years have witnessed sustained growth in “pet-related” content across online video platforms. Humans demonstrate a higher preference for pet videos compared to most other video categories (X. Zhang et al., 2024; Si et al., 2025). Chronic indirect media exposure to pets appears to confer mental health benefits (S. Li et al., 2025), though the cognitive mechanisms remain underexplored due to limited empirical investigations. This study therefore employs three experiments to investigate how viewing pet videos affects emotional–cognitive processing, aiming to elucidate the cognitive mechanisms through which media-based pet exposure enhances mental health.

Multiple theoretical frameworks have been proposed to explain the mood-enhancing effects of pets. Bowlby’s attachment theory posits that individuals form robust social bonds due to the security and support provided by specific entities (Bowlby, 2008). Recent research has expanded attachment theory to encompass non-human targets, such as pets, that can provide analogous feelings of security and support (Keefer et al., 2014). Attachment exhibits significant correlations with health outcomes, including emotional regulation, stress responses, perceived social support, and health-related behaviors (Taylor et al., 2024). Many pet owners identify pets as crucial sources of emotional support, with heightened attachment levels potentially mediating their psychological benefits (Langston, 2014). Additionally, the biophilia hypothesis proposes an innate human predisposition to affiliate with animals (Wilson, 1984). This inclination may confer adaptive advantages at the individual level (Mormann et al., 2011). The baby schema theory maintains that facial features like large eyes, small noses, and rounded contours elicit caregiving responses and positive affect in humans (Glocker et al., 2009). These positive emotional states evoked by pets contribute to enhanced psychological well-being. While these theoretical models collectively elucidate mechanisms underlying pets’ mental health impacts, whether similar pathways mediate the effects of media-mediated indirect exposure remains unexplored.

Individuals respond to media content with reactions akin to real-life experiences (Mancuso et al., 2023; Z. Zhang et al., 2024). Lisa et al. (2004) demonstrated that participants exposed to the Cyberball task exclusion exhibited equivalent distress responses whether the exclusion originated from humans or computer agents. The content of media significantly affects the emotion experience of users when they use media and, in turn, influences their emotions and behaviors in real life (Z. Zhang et al., 2024). It suggests that media consumption of pet content may similarly impact psychological well-being.

Conversely, numerous studies have demonstrated that viewing pet videos and images alleviates stress and enhances positive affect (Ein et al., 2022, 2023; Na & Dong, 2023; Lavan et al., 2023). For instance, Ein et al. (2023) observed that participants who viewed pet videos prior to stress-inducing tasks exhibited attenuated stress responses during subsequent task performance. Lavan et al. (2023) reported significant increases in subjective well-being and physiological relaxation following three-minute exposures to puppy videos. These findings collectively indicate that indirect contact via visual media effectively modulates emotional states. This raises the critical question: Can sustained media exposure to pet content confer enduring mental health benefits?

Myrick (2015) assessed the mental health of individuals who watch cat videos and view cat images online through questionnaires. The results demonstrated that exposure to such content enhances positive emotions and reduces negative emotions, suggesting that media-based interaction with pets may benefit mental health. However, S. Li et al. (2025) compared the mental health of three groups: pet owners, non-owners, and people who frequently watch pet-related content online. The results found that pet owners exhibited significantly better mental health than non-owners. There was no significant difference in mental health between frequent online consumers of pet content and pet owners. While the frequent online consumers also showed no significant mental health advantage over non-owners. Moreover, the mechanisms by which pet ownership influences mental health differ from those linked to online exposure to pet content. S. Li et al. (2025) suggest that whether long-term media exposure to pet content can improve mental health remains to be explored.

While exposure to pet imagery and videos may enhance short-term emotional states, research on long-term outcomes remains inconclusive, and self-reported measures often fail to confirm causal relationships. Consequently, experimental research into the effects of pet media on human psychology holds critical scientific importance.

Aberrant emotional processing biases are prevalent in individuals with emotional disorders (W. Lin et al., 2021; Wei et al., 2024; Zhou & Shen, 2024). Targeted interventions addressing these biases demonstrate efficacy in mitigating symptoms of emotional disorders (MacLeod & Clarke, 2015; Gao et al., 2022; J. Lin et al., 2024). If exposure to pet media influences cognitive–emotional biases, prolonged engagement with such content may alter mental health outcomes through persistent modifications in emotional processing.

Human faces exhibit high social salience and distinct neural processing pathways (Y. Li et al., 2024; J. Yang et al., 2024; M. Yang et al., 2024). For example, individuals tend to prioritize the processing of objects with social significance, such as faces (Y. Li et al., 2024). The mental health benefits of pets are mediated by their capacity to fulfill social support needs (S. Li et al., 2025). We hypothesize that pet media may modulate facial emotion processing through differential cognitive–emotional engagement. This study uses emotional facial stimuli to examine how exposure to pet videos alters emotion processing, thereby elucidating potential pathways linking media-based exposure to pets with mental health outcomes.

## 2. Experiment 1: The Impact of Pet Videos on Attentional Bias Towards Emotional Faces

Individuals with emotional disorders exhibit clinically distinct attentional processing patterns compared to healthy controls (MacLeod & Clarke, 2015). Building on evidence that pets elicit unique emotion-regulatory effects compared to nonsocial positive stimuli—potentially mediated by their social salience (S. Li et al., 2025)—this study rigorously compares how pet videos versus nonsocial positive stimuli (e.g., scenery videos) modulate attentional engagement with emotional faces, assessing both quantitative differences and qualitative mechanistic distinctions. The experimental paradigm investigates how exposure to three video categories (pet, scenery, and neutral) dynamically shapes attentional allocation to faces varying in emotional valence (positive, neutral, and negative).

Hypothesis 1 was that the attentional bias of the participants towards positive faces showed significant differences among the three conditions. The attentional bias in the pet condition was higher than that in the scenery condition, and the attentional bias in the scenery condition was higher than that in the neutral condition. Hypothesis 2 was that the attentional bias of the participants towards negative faces also showed significant differences among the three conditions. The attentional bias in the pet condition was lower than that in the scenery condition, and the attentional bias in the scenery condition was lower than that in the neutral condition.

### 2.1. Method

#### 2.1.1. Participants

A medium effect size of f = 0.25 was used to conduct a power analysis with G*power 3.1. The estimated sample size needed was at least 34 participants to achieve 80% power and detect the effect given an α level of 0.05. The current experiment recruited 36 college students with normal or corrected-to-normal vision (23 females, aged 19.81 ± 1.49 years). None of these participants had participated in other experiments from this research.

#### 2.1.2. Experimental Materials

(1) Videos

All stimuli were sourced from publicly available online repositories and standardized to 10 min silent clips. Pet stimuli exclusively depicted cats or dogs, excluding other species or human interaction. Scenery stimuli comprised natural landscapes devoid of fauna, humans, or anthropogenic elements. Neutral control stimuli consisted of digitally simulated film scratches, with white letters K and L presented centrally for 5 s at the 4 min and 8 min marks. A pilot study was conducted prior to the main experiment, in which 30 participants evaluated the emotional valence of 3 videos on a 7-point scale, with 7 being the most positive. Pilot experiment participants did not take part in any formal experiment. The purpose of the pilot study was to demonstrate that there is no significant difference in valence between pet videos and scenery videos, and that the valence of both is higher than that of neutral videos. A repeated-measures ANOVA with 3 (video type: pet, scenery and neutral) factors revealed that the main effect of valence was significant [*F*(2, 58) = 102.304, *p* < 0.001]. A post hoc test revealed that there was no significant difference between pet and scenery [*p* = 0.702], the pet was higher than the neutral [*p* < 0.001], and the scenery was significantly higher than the neutral [*p* < 0.001].

All animals shown in the videos met established indicators of positive animal welfare (e.g., expression of natural behaviors and positive mental states), following the consensus framework proposed by Rault et al. (2025), to ensure compliance with ethical research standards.

(2) Faces

Twenty-four negative, 24 positive, and 48 neutral facial stimuli were sourced from the Chinese Facial Affective Picture System (CFAPS; Y. Wang & Luo, 2005), with equal gender distribution (male/female). Each trial paired one emotional face (negative/positive) with a neutral face, forming 24 negative-neutral and 24 positive-neutral dyads. Neutral faces were counterbalanced across left/right positions within pairs to control for spatial bias. Trial types (consistent/inconsistent valence-side congruence) were equally distributed. Facial stimuli measured 7 cm (width) × 8 cm (height) on the display. The center-to-center distance between paired stimuli was fixed at 4 cm.

#### 2.1.3. Experimental Procedure

The experimental procedure was programmed using Presentation 0.71 software. The participants sat in a seat in a soundproof room to complete the experiment. The background of the monitor was black, and the monitor was placed 80 cm away from the participant.

Participants completed three experimental sessions spaced at least three days apart. Identical procedures were followed across all sessions, with participants viewing distinct video categories in each session. A Latin square design was implemented to counterbalance video presentation order across participants.

Participants first completed a dot-probe task. Each trial comprised (1) a central fixation cross (+) for 500 ms; (2) a face pair displayed for 500 ms; (3) a 50 ms blank screen; (4) a probe (*) appearing for 2000 ms at one face location. Participants responded via keypress (F = left, J = right) to indicate probe position. Trials were classified as valence-congruent (probe replaced emotional face) or valence-incongruent (probe replaced neutral face). Congruent/incongruent trials and left/right probe positions were counterbalanced across conditions. (5) a 1000 ms inter-trial interval before the next trial.

After completing the dot-probe task, participants viewed a video, subsequently evaluated its valence using a 7-point scale, and responded to two attention-check questions. Any incorrect answer led to experiment termination and data exclusion (in fact, no participant was excluded). For the pet animal condition, questions included “Was a border collie present in the video?” and “Was a Garfield-cat depicted in the video?” Scenery condition queries were “Did the video showcase a starry sky?” and “Was a snowy landscape featured in the video?” Neutral condition questions asked, “Was the letter K present in the video?” and “Was the letter G displayed in the video?”.

Finally, participants performed the dot-probe task again, with the same materials and procedure as the first task.

#### 2.1.4. Data Analysis

Analyses were performed using SPSS 26.0. A measure of attention bias index was computed based on the work of MacLeod and Mathews (1988): Attention Bias Index (ABI) = *RT_incon_* − *RT_con_*. Specifically, the attentional bias index was calculated by subtracting reaction times in congruent trials from reaction times in incongruent trials. A positive attentional bias index indicates that reaction times in incongruent trials were longer than those in congruent trials, suggesting faster responses in congruent trials and thus the presence of an attentional bias toward emotional stimuli. In this case, participants responded more rapidly to probes appearing at the location of emotional stimuli, whereas probes presented at the location of neutral stimuli required longer reaction times due to attentional reorienting. An attentional bias index of zero indicates no difference in reaction times between congruent and incongruent trials, reflecting the absence of attentional bias. A negative attentional bias index indicates shorter reaction times in incongruent trials than in congruent trials, suggesting faster responses in incongruent trials and reflecting attentional avoidance of emotional stimuli. In this case, participants responded more rapidly to probes appearing at the location of neutral stimuli and more slowly to probes appearing at the location of emotional stimuli, indicating avoidance of emotional information.

This study analyzed the difference between pre- and post-test attentional bias indexes as the dependent variable. Attention Bias Index Difference = ABIpre − ABIpost. The change in attentional bias was computed as the attentional bias index before video viewing minus the attentional bias index after video viewing. The more positive value indicated less post-video bias towards emotional stimuli. The attentional bias index (difference value) and video emotion valence were analyzed using a repeated-measures ANOVA, with post hoc comparisons conducted using the LSD test and the Bonferroni correction applied to adjust for multiple comparisons.

### 2.2. Results

**Figure 1:**
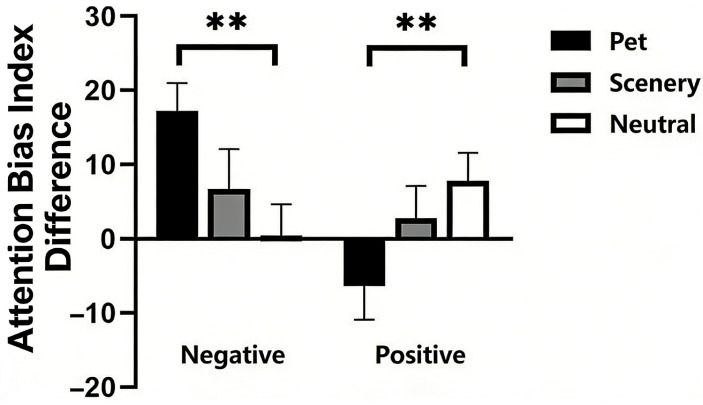
Attention bias index difference. Attention bias index difference = ABIpre – ABIpost. The attentional bias index difference was defined as the attentional bias index before minus after video viewing, with more positive values indicating reduced attentional bias toward emotional stimuli. ** *p* < 0.01. Error bars represent means ± SEM.

A repeated-measures ANOVA on the attention bias index with emotion face (positive, negative) and video type (pet, scenery and neutral) as within-subject variables revealed a significant interaction effect between emotion face and video type [*F*(2, 70) = 5.947, *p* < 0.01, *ηp*^2^ = 0.145]. Simple effects analyses revealed pets were significantly higher than neutral [*p* < 0.01] in the negative condition. There was no significant difference between scenery and neutral [*p* = 0.347]. The difference between pet and scenery was also not significant [*p* = 0.113]. In the positive condition, pets were significantly lower than neutral [*p* < 0.01]. There was no significant difference between scenery and neutral [*p* = 0.324]. The difference between pets and scenery was also not significant [*p* = 0.160]. None of the main effects were significant (Figure 1 and Table 1).

A repeated-measures ANOVA on emotion valence of video with emotion valence (pet, scenery and neutral) as within-subject variables revealed a significant main effect [*F*(2, 70) = 99.588, *p* < 0.001, *ηp*^2^ = 0.740]. Post hoc tests showed that neutral was significantly lower than that for pet [*p* < 0.001] and scenery [*p* < 0.001]. There was no significant difference between pet and scenery [*p* = 0.474].

### 2.3. Discussion

This study investigated how viewing pet videos influences subsequent emotional attentional bias. Findings revealed that after watching pet videos, participants exhibited a significant reduction in attentional bias towards negative emotional faces and a notable increase towards positive ones. In contrast, scenery videos showed no such effects.

Posner and Petersen (1990) divide the human attention system into several subsystems, two of which are relevant in the present context: orienting toward stimuli and disengaging from stimuli. The attenuated attentional bias toward negative-valenced facial stimuli suggests that pet-related video exposure decreases attentional maintenance on negative faces (greater orientation toward neutral faces) or facilitates disengagement from such stimuli (easier to disengage attention from negative faces) or the coexistence of both effects. Conversely, the heightened bias toward positive-affective cues reflects a reversal in cognitive prioritization patterns.

In the negative face condition, the attention bias index of the pet condition was significantly higher than that of the neutral condition. This result indicates that after the participants watched the pet videos, the time they spent looking at the negative faces significantly decreased. Watching pet videos can reduce people’s attention bias towards negative faces. In the positive face condition, the attention bias index of the pet condition was significantly lower than that of the neutral condition. This result also shows that watching pet videos can increase people’s attention bias towards positive faces.

While both the pilot and formal experiments demonstrated that pet and scenery videos had significantly higher valence than neutral videos, with no significant difference between them, their effects on attentional bias towards emotional faces differed. The pet videos used in the present study featured cats and dogs, which are the animals most commonly kept as companion animals in daily life. Unlike scenery videos, cats and dogs share close emotional bonds with humans. Liu et al. (2025) reported that viewing images of companion animals (cats and dogs) specifically activates brain regions associated with human attachment, as well as neural circuits involved in memory, emotion, and attachment-related cognitive processing. These findings suggest that viewing images of companion animals engages the human socio-emotional system. Furthermore, previous research has demonstrated that humans can form attachment relationships with pets, which provide emotional support and comfort (Keefer et al., 2014).

Therefore, we propose that the attentional bias–modulating effect of pet videos may not be solely attributable to positive mood induction but could also be related to the social support they provide. The social support from pets in videos may influence participants, altering their attentional bias towards socially attributed emotional faces. Conversely, scenery videos, although also inducing positive emotions, lack this effect. This indicates that pet videos exert a distinctive influence on human emotion regulation.

## 3. Experiment 2: The Impact of Pet Videos on the Valence Assessment of Emotional Faces

Experiment 1 demonstrates that viewing pet videos induces a specific alteration in participants’ attentional bias towards emotional faces, yet the underlying mechanism remains unclear. A potential explanation could be a shift in participants’ perception of facial emotion valence. However, if this attentional bias change is indeed attributable to altered emotional valence perception, when neutral and emotional stimuli are concurrently presented in experiments, it becomes impossible to discern whether the attentional bias change results from altered perception of emotion in neutral faces or emotional faces. Consequently, Experiment 2 aims to directly examine whether participants’ subjective perception of emotional face valence changes following exposure to three video types, thereby further investigating the impact of pet information on emotional cognitive processing.

Hypothesis 3 was that the valence of faces in the pet condition was higher than that in the scenery condition, and the valence of faces in the scenery condition was higher than that in the neutral condition.

### 3.1. Method

#### 3.1.1. Participants

A medium effect size of f = 0.25 was used to conduct a power analysis with G*power 3.1. The estimated sample size needed was at least 34 participants to achieve 80% power and detect the effect given an α level of 0.05. The current experiment recruited 36 college students with normal or corrected-to-normal vision (25 females, aged 20.94 ± 1.19 years). None of these participants had participated in other experiments from this research.

#### 3.1.2. Experimental Materials

(1) Videos

Same as Experiment 1.

(2) Faces

Thirty-two negative, 32 neutral, and 32 positive faces were selected from the Chinese Facial Affective Picture System (CFAPS). There were equal numbers of male and female faces. Face pictures were presented at the center of the screen with a size of 7 cm by 8 cm.

#### 3.1.3. Experimental Procedure

The experiment was conducted in the same sound-attenuated laboratory. Participants completed the experiment in three separate sessions, each spaced at least three days apart, during which they viewed three categories of videos (pet, scenery, and neutral) and completed the corresponding valence evaluation tasks. The order of video categories was counterbalanced across participants using a Latin square design to control for order effects.

At the beginning of each session, participants were seated 80 cm from the display monitor. The experimental procedure was programmed and presented using Presentation 0.71 software on a monitor with a refresh rate of 60 Hz.

Each experimental session consisted of three phases. First, participants watched a 10 min silent video corresponding to the assigned condition. Immediately afterward, they completed an emotional face valence evaluation task. In each trial, a white fixation cross was presented at the center of the screen for 500 ms, followed by the presentation of an emotional face (positive, negative, or neutral), which remained on the screen until a response was made. Participants were instructed to rate the valence of the face using numeric keys on the keyboard (1–7; 1 = “most negative”, 7 = “most positive”). Upon response, the stimulus disappeared, followed by a randomly jittered blank interval of 1100–1500 ms before the next trial. The task comprised 96 trials presented in random order, including 32 positive, 32 negative, and 32 neutral faces.

Finally, participants answered two attention-check questions related to the viewed video and rated the valence of the video itself using a 7-point scale (1 = very negative, 7 = very positive).

The experimental environment, apparatus, video materials, and attention-check procedures were identical to those used in Experiment 1. The primary difference was that the dot-probe task employed in Experiment 1 was replaced by the valence evaluation task in the present experiment.

#### 3.1.4. Data Analysis

Analyses were performed using SPSS 26.0. The emotional valence of facial stimuli and videos was analyzed using a repeated-measures ANOVA, with post hoc comparisons conducted using the LSD test and Bonferroni correction applied to adjust for multiple comparisons.

### 3.2. Results

**Figure 2:**
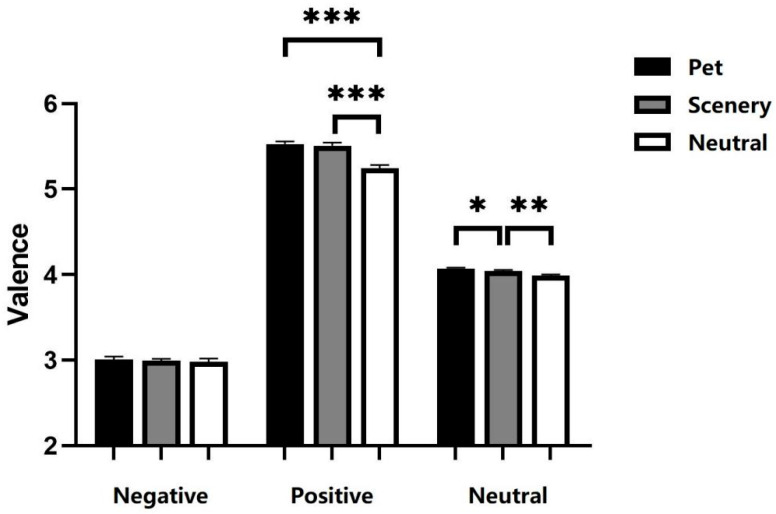
Perceived valence of emotional faces. * *p* < 0.05, ** *p* < 0.01, *** *p* < 0.001. Error bars represent means ± SEM.

A repeated-measures ANOVA on emotion valence with emotion face (positive, negative, neutral) and video type (pet, scenery and neutral) as within-subject variables revealed a significant main effect of emotion face [*F*(2, 70) = 2644.913, *p* < 0.001, *ηp*^2^ = 0.987] and video type [*F*(2, 70) = 33.667, *p* < 0.001, *ηp*^2^ = 0.490]. The interaction between emotion face and video type was significant [*F*(4, 140) = 11.809, *p* < 0.001, *ηp*^2^ = 0.252]. Simple effects analyses revealed neutral were significantly lower than pet [*p* < 0.001] and scenery [*p* < 0.001] in the positive condition. In the negative condition, there was no significant main effect of video type [*p* = 0.642]. In neutral conditions, pets were significantly higher than scenery [*p* < 0.05]. Scenery was significantly higher than neutral [*p* < 0.01] (Figure 2).

A repeated-measures ANOVA on emotion valence of video with emotion valence (pet, scenery and neutral) as within-subject variables revealed a significant main effect [*F*(2, 70) = 199.894, *p* < 0.001, *ηp*^2^ = 0.851]. Post hoc tests showed that neutral was significantly lower than that for pet [*p* < 0.001] and scenery [*p* < 0.001]. There was no significant difference between pet and scenery [*p* = 0.291].

To explore whether the change in attentional bias towards positive emotional faces in Experiment 1 was caused by a change in valence perception. A repeated-measures ANOVA on emotion valence with emotion face (positive, neutral) and video type (pet, neutral) as within-subject variables was conducted. The interaction between emotion face and video type was significant [*F*(1, 35) = 19.534, *p* < 0.001, *ηp*^2^ = 0.358]. The disparity between pet stimuli and neutral videos was more pronounced in positive facial expression conditions compared to neutral facial expression conditions.

### 3.3. Discussion

This study investigated how viewing pet videos influences subsequent perception of facial emotional valence. Results demonstrated that for negative faces, no significant valence differences existed among the three video types. For positive faces, pet and scenery videos exhibited significantly higher valence than neutral videos, with no significant difference between pet and scenery videos. For neutral faces, pet valence was significantly higher than scenery, which was significantly higher than neutral. The participant gave a higher score for the facial valence, indicating that he perceived the face as more positive. Pets exerted a greater influence on valence perception of positive emotional faces compared to neutral ones.

The results of this experiment show that watching emotional videos can significantly influence subsequent valence perception of emotional faces. A recent hypothesis, called the affective realism hypothesis, proposes that affective feelings, as integral properties of individuals’ perceptual experiences, akin to experiences of hue and brightness, influence target perception (Anderson et al., 2012; Kring et al., 2014; Gao et al., 2022). Events encountered after an arousing emotional experience may be processed through specific emotional filters, thus “coloring” the perception or evaluation of the event (Grupe et al., 2018). Therefore, the emotions experienced by the participants while watching the video will affect their perception of emotions during the subsequent face processing.

Emotional videos influence various emotional face types distinctly. Positive emotional videos can enhance the valence perception of neutral and positive faces but have no significant effect on negative ones. This might be because negative stimuli hold survival significance (W. Lin et al., 2021), making them less susceptible to influence from other emotional stimuli.

More importantly, pet videos demonstrated specificity in influencing facial emotion perception. For neutral faces, pet videos had a significantly greater impact on emotion perception than scenery videos. This could be attributed to pets’ social attributes influencing participants’ emotion perception. Xu et al. (2016) used emotionally and socially charged linguistic information as a background to investigate the impact of emotion and sociality on neutral face processing. They discovered that face processing is affected by the interplay of emotion and sociality. In line with previous research, this study suggests that pets’ social attributes can specifically influence people’s emotional processing of faces with similar attributes.

## 4. Experiment 3: The Impact of Pet Animal Videos on the Valence Assessment of Emotional Words

The consistent results of Experiment 1 and Experiment 2 demonstrate that pet videos can specifically influence attentional bias toward emotional faces and emotional perception. This effect is likely attributable to the social attributes of pets and faces. If the perception target lacks social attributes, pet videos may not exert specific effects on emotional perception. Consequently, this experiment employs emotional words as the perception target, investigating the impact of emotional videos on valence assessment of emotional words.

Hypothesis 4 was that there was no significant difference in the valence of the positive words, negative words and neutral words among the three conditions.

### 4.1. Method

#### 4.1.1. Participates

A medium effect size of f = 0.25 was used to conduct a power analysis with G*power 3.1. The estimated sample size needed was at least 34 participants to achieve 80% power and detect the effect given an α level of 0.05. The current experiment recruited 36 college students with normal or corrected-to-normal vision (26 females, aged 21.22 ± 1.22 years). None of these participants had participated in other experiments from this research.

#### 4.1.2. Experimental Materials

(1) Videos

Same as Experiment 1.

(2) Words

Thirty-two negative words, 32 neutral words, and 32 positive words were selected from the Chinese Affective Words System (Y. Wang et al., 2008). All words were two-character nouns. The text was presented on the screen with a size of 4.5 cm × 2 cm. The face was located at the center of the screen.

#### 4.1.3. Experimental Procedure

The experimental procedure of Experiment 3 was identical to that of Experiments 1 and 2 in terms of environment, apparatus, and overall design. Participants visited the laboratory on three separate occasions, with intervals of at least three days between sessions, to complete viewing of the three video categories (pet, scenery, and neutral) followed by an emotional word valence evaluation task. The order of video categories was counterbalanced across participants using a Latin square design.

Each session was conducted in a sound-attenuated room, with participants seated 80 cm from the display monitor. The experimental program was implemented using Presentation 0.71 software and presented on a monitor with a refresh rate of 60 Hz.

Each session proceeded as follows. First, participants watched a 10 min silent video corresponding to the assigned condition. Immediately thereafter, they completed an emotional word valence evaluation task. In each trial, a white fixation cross (+) was presented at the center of the screen for 500 ms, followed by the presentation of a two-character emotional word (positive, negative, or neutral), which remained on the screen until a response was made. Participants were instructed to judge the emotional valence of each word as quickly and accurately as possible using numeric keys on the keyboard (1–7; 1 = “most negative,” 7 = “most positive”). Upon response, the word disappeared, followed by a randomly jittered blank interval of 1100–1500 ms. The word evaluation task comprised 96 trials presented in random order, including 32 positive, 32 negative, and 32 neutral words.

After completing the task, participants answered two attention-check questions related to the viewed video (e.g., in the pet video condition, “Was Garfield the cat shown in the video?”) and rated the emotional valence of the video itself using a 7-point scale (1 = very negative, 7 = very positive).

The experimental environment, phased procedure, video materials, Latin square counterbalancing, and attention-check procedures were strictly identical to those used in Experiments 1 and 2. The primary distinction was that the perceptual targets in Experiment 2 (emotional faces) were replaced with emotional words in the present experiment, allowing for examination of differences in the effects of pet videos on the processing of social versus non-social emotional stimuli.

#### 4.1.4. Data Analysis

Analyses were performed using SPSS 26.0. The emotional valence of emotional words and videos was analyzed using a repeated-measures ANOVA, with post hoc comparisons conducted using the LSD test and Bonferroni correction applied to adjust for multiple comparisons.

### 4.2. Results

**Figure 3:**
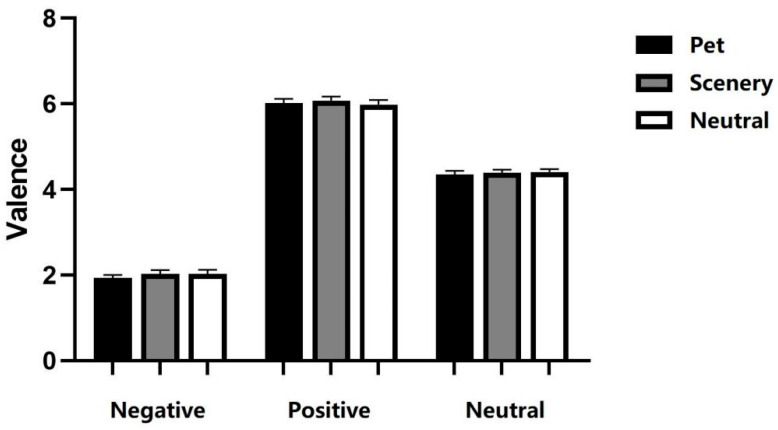
Perceived valence of emotional words. Error bars represent means ± SEM.

A repeated-measures ANOVA on emotion valence with emotion word (positive, negative, or neutral) and video type (pet, scenery or neutral) as within-subject variables revealed a significant main effect of emotion word [*F*(2, 70) = 711.714, *p* < 0.001, *ηp*^2^ = 0.953]. There was no significant main effect of video type [*F*(2, 70) = 1.967, *p* = 0.147, *ηp*^2^ = 0.053] or interaction effect [*F*(4, 140) = 0.511, *p* = 0.728, *ηp*^2^ = 0.014] (Figure 3).

A repeated-measures ANOVA on emotion valence of video with emotion valence (pet, scenery and neutral) as within-subject variables revealed a significant main effect [*F*(2, 70) = 68.844, *p* < 0.001, *ηp*^2^ = 0.663]. Post hoc tests showed that neutral was significantly lower than that for pet [*p* < 0.001] and scenery [*p* < 0.001]. There was no significant difference between pet and scenery [*p* = 0.133].

### 4.3. Discussion

This study investigated how viewing pet videos affected subsequent emotional valence perception of words. The results indicated that none of the videos influenced emotional valence perception.

Numerous studies have demonstrated distinct processing mechanisms between text and images in humans, supported by behavioral (W. Lin et al., 2022) and neuroimaging evidence (Vandenberghe et al., 1996; Reisch et al., 2020; W. Lin et al., 2023). For example, emotional effects elicited by words emerge selectively in late-stage semantic processing, while facial emotional processing dominates early sensory stages (Rellecke et al., 2011; Reisch et al., 2020). Therefore, the valence ratings of emotional words may exhibit lower sensitivity to video-induced modulation compared to facial stimuli.

It should be noted that one of the primary aims of the present experiment was to examine whether the modulatory effect of pet videos on emotional processing depends on the social attributes of the stimuli (e.g., faces used in Experiments 1 and 2). The results showed that pet videos did not modulate the valence perception of non-social emotional words, suggesting that their effects may be more sensitive to stimuli with social attributes. However, this null effect may also be attributable to systematic differences between words and faces in terms of emotional immediacy, sensory modality, or depth of processing. Future research could further clarify the boundary conditions and cognitive mechanisms underlying the effects of pet videos on emotional processing by directly comparing different types of social and non-social stimuli.

## 5. General Discussion

This study investigated how pet videos influence emotion processing through three experiments. The results demonstrated that viewing pet videos significantly modulated subsequent attentional bias toward emotional faces by enhancing valence perception of both positive and neutral facial stimuli.

Numerous studies have demonstrated that neural activity and functional connectivity in the brain undergo changes following exposure to emotional stimuli (Eryilmaz et al., 2011; Tambini et al., 2016; Qiao-Tasserit et al., 2024). Tambini et al. (2016) revealed that participants’ brains remained emotionally engaged for a period after such exposure, influencing subsequent encoding of emotional stimuli. Some studies even reported increased activity in brain regions such as the anterior cingulate cortex and inferior parietal lobe after watching emotionally charged film clips, compared to activity levels during clip viewing (Eryilmaz et al., 2011). These findings suggest that human emotions and related neural activities remain active for some time after emotional stimulus offset, thereby affecting subsequent cognitive processing. Consequently, the emotional state induced by pet videos did not immediately dissipate but persisted, resulting in altered emotional attention bias and perception in subsequent tasks.

Research findings indicate that pet videos primarily influence emotional face processing by enhancing perceived valence for positive and neutral faces. While Experiment 1 demonstrated that pet videos reduced attentional bias toward negative faces, Experiment 2 revealed this effect was mediated through enhanced valence perception of paired neutral faces, rather than directly influencing attention to negative faces.

In Experiment 2, pet videos enhanced valence perception of both neutral and positive faces, with a significantly stronger enhancement for positive faces compared to neutral ones. Stimuli with higher valence are known to attract greater attentional resources (Carretié et al., 2001). This accounts for why pet videos in Experiment 1 increased participants’ attentional bias toward positive faces. This effect may stem from a protective “positivity bias” (Grupe et al., 2018), wherein individuals exhibit heightened sensitivity to positive information. Such a positivity bias has been widely documented across domains, including media (Ng et al., 2023), face processing (Zhao et al., 2017), memory (Ünal & Besken, 2020; Ferguson et al., 2023), and language (Dodds et al., 2014).

Results from Experiments 1–3 demonstrate that pet videos specifically modulate emotional face processing. People primarily keep pets for emotional support (X. Zhang et al., 2024), and media content can produce effects analogous to real-life interactions (Mancuso et al., 2023; Z. Zhang et al., 2024). When viewing pet videos, participants may experience emotional support akin to real-life interactions. Emotional support has been shown to significantly modulate attentional bias toward emotional faces (Dewitte & De Houwer, 2008). Therefore, transient emotional support induced by the videos leads to temporary alterations in emotion processing. However, it should be noted that the present experiment did not directly measure participants’ perceived social support while viewing the videos. Therefore, it remains unclear whether the effects of pet videos arise from simulated social support and the fulfillment of affiliation needs, or from a more general activation of attentional and affective systems related to living entities. Further investigation is required to disentangle these possibilities.

This study demonstrates that viewing pet videos significantly modulates subsequent emotion processing. Therefore, we propose that the regulatory effects of long-term exposure to pet-related media content on mental health may operate through two distinct pathways.

First, the impact of pet videos on subsequent emotion processing may not be limited to the task period and could last for days or longer. In Grupe et al. (2018), participants were presented with three valence emotional pictures paired with neutral faces. Three days later, their liking and memory for the neutral faces were tested, revealing that both were influenced by the emotional pictures shown three days earlier. The authors suggest that affective information may spill over from preceding and unrelated emotional events in a way that can color the perception or evaluation of unrelated social stimuli. Therefore, regularly watching pet videos may color a large amount of emotional information in people’s lives more positively, especially social information, and this positivity can persist in daily life, contributing to better mental health.

Second, the mental health effects of frequent exposure to pet videos may parallel those of positive psychological interventions (PPIs). PPIs are defined as repeated engagement in activities designed to elicit positive emotions, thereby fostering positive emotional states, behaviors, or cognitive patterns in individuals (Sin & Lyubomirsky, 2009), with the ultimate goals of enhancing well-being and alleviating mental disorders. Established PPIs include written positive experiences (Burton & King, 2004), positive future thinking (Peters et al., 2010), and positive self-bias training (L. Wang et al., 2023). The emotional support derived from pets may potentiate the efficacy of such interventions, particularly in enhancing social functioning. It is noteworthy that although the pet videos used in this study were sourced from publicly available online platforms, future applications of such content in formal mental health interventions or research should adhere strictly to non-invasive, animal welfare–oriented ethical guidelines. The production of videos should not pursue human psychological benefits as the sole objective; rather, it should also promote positive human–animal interactions and shared well-being, ensuring that animals remain in a natural, comfortable, and stress-free state throughout recording (Rault et al., 2025). Such practices are not only an ethical requirement for scientific research but also constitute a foundation for developing sustainable and responsible human–animal interactive media.

In summary, long-term exposure to pet videos and images through media may influence mental health via modulation of emotion processing. Our findings provide empirical evidence for elucidating the relationship between media content and human mental health, particularly highlighting the beneficial effects of media-delivered positive information.

This study has several limitations. First, the pet videos used in the experiment lacked real interactions between humans and animals, which limits the extent to which their effects can be directly equated with the social support derived from actual human–animal interactions. Future research should compare the effects of interactive versus non-interactive pet content. Second, we did not assess participants’ subjective experience of social support while watching the videos. Future studies could combine such self-report measures with neuroimaging techniques (e.g., fMRI, EEG) to more directly elucidate the underlying cognitive and neural mechanisms. In addition, because participants’ pet ownership status was not the primary focus of this study, data regarding this variable were neither collected nor analyzed. Future research could consider including participants’ pet ownership and attitudes toward companion animals as potential variables for further investigation. Finally, this study employed a dot-probe task to measure attentional bias. However, it should be noted that the dot-probe task has been widely criticized for its low reliability (Kappenman et al., 2014). Future research could adopt more reliable experimental paradigms or combine eye-tracking and EEG techniques to validate the present findings and further clarify their cognitive mechanisms. Future investigations could employ longitudinal experimental designs or cognitive neuroscience techniques to further delineate the mechanisms underlying the mental health impacts of chronic media consumption of pet-related content.

## 6. Conclusions

Our results demonstrate that viewing pet videos decreases attentional bias toward subsequent negative emotional faces while increasing it toward positive ones. This effect stems from enhanced valence perception of subsequent positive and neutral faces. The modulatory effect on emotional faces is specific to pet videos compared to non-social positive ones, attributable to their emotional support properties.

## Figures and Tables

**Table 1:** Reaction times and attention bias indices in the dot-probe task for each video condition and face valence. Standard deviation is presented in brackets.

	Face Valence	Pre-Video Viewing (ms)		Post-Video Viewing (ms)		Attention Bias Index (ABI) (ms)		Attention Bias Index Difference (ms)
		**RTincon**	**RTcon**	**RTincon**	**RTcon**	**ABIpre**	**ABIpost**	**ABIpre-ABIpost**
Pet	Negative	372.26 (86.33)	354.55 (86.17)	319.98 (67.69)	319.48 (63.99)	17.71 (31.48)	0.50 (41.72)	17.20 (22.30)
	Positive	352.75 (83.91)	344.70 (77.20)	342.01 (95.61)	327.58 (74.25)	8.05 (41.00)	14.43 (49.30)	−6.38 (27.41)
Scenery	Negative	377.11 (90.03)	360.89 (93.48)	328.80 (70.19)	319.21 (52.69)	16.23 (33.31)	9.59 (47.72)	6.64 (32.27)
	Positive	358.65 (82.18)	349.48 (72.04)	325.47 (66.21)	319.02 (49.27)	9.12 (22.87)	6.46 (34.71)	2.71 (26.33)
Neutral	Negative	422.62 (157.86)	408.59 (154.47)	374.12 (118.96)	360.47 (115.10)	14.03 (38.92)	13.65 (50.89)	0.38 (25.64)
	Positive	407.49 (161.39)	402.07 (164.53)	356.67 (109.94)	359.03 (107.94)	5.42 (30.00)	−2.36 (38.42)	7.78 (22.64)

## Data Availability

The datasets used during the current study are available from the corresponding author.
